# Engagement in sexual healthcare and STI/HIV burden of first- and second-generation migrant and Western-born female sex workers in the Netherlands: A retrospective cohort study

**DOI:** 10.1016/j.jmh.2024.100281

**Published:** 2024-10-31

**Authors:** C.M.M. Peters, Y.J. Evers, C.J.G. Kampman, M.J. Theunissen–Lamers, M.A.M. van den Elshout, N.H.T.M. Dukers-Muijrers, C.J.P.A. Hoebe

**Affiliations:** a Department of Social Medicine, Care and Public Health Research Institute (CAPHRI), Maastricht University/Maastricht UMC+, PO Box 616, 6200 MD Maastricht, the Netherlands; b Department of Sexual Health, Infectious Diseases and Environmental Health, Living Lab Public Health Mosa, South Limburg Public Health Service, PO Box 33, 6400 AA, Heerlen, the Netherlands; c Public Health Service Twente, PO Box 1400, 7511 JM Enschede, the Netherlands; d Department of Sexual Health, Public Health Service Brabant-Zuidoost, P.O Box 8684, 5605 KR Eindhoven, the Netherlands; e Department of Infectious Diseases, Public Health Service of Amsterdam, Amsterdam, the Netherlands; f Department of Sexual Health, Public Health Service of the Utrecht Region, Utrecht, the Netherlands; g Department of Health Promotion, Care and Public Health Research Institute (CAPHRI), Maastricht University/Maastricht UMC+, PO Box 616, 6200 MD Maastricht, the Netherlands; h Department of Medical Microbiology, Infectious Diseases and Infection Prevention, Care and Public Health Research Institute (CAPHRI), Maastricht University Medical Centre (MUMC+), PO Box 5800, 6202 AZ Maastricht, the Netherlands

**Keywords:** Female sex workers, Sexually transmitted infections, Healthcare-seeking behaviour, Migration, Migrant sex workers, Western Europe

## Abstract

**Introduction:**

Scarcely available European studies suggest that migrant female sex workers (FSW) have a higher likelihood of sexually transmitted infections (STI) but a lower likelihood of HIV compared to non-migrant FSW. This study assessed demographics, STI/HIV burden, and engagement in sexual healthcare among first-generation (FGM) and second-generation (SGM) migrant FSW versus Western-born FSW.

**Methods:**

This large retrospective cohort study included 27,532 Dutch STI clinic consultations from 11,363 individual FSW between 2016 and 2021. STI diagnoses (chlamydia/gonorrhoea/ infectious syphilis/infectious hepatitis B/HIV) in the first consultation were compared using Chi-squared test. Logistic regression adjusting for age, urbanity and sexual behaviour assessed associations between migration status and STI diagnoses. Incidence of repeat consultation was compared between migration groups using Cox proportional hazards regression, adjusting for age and STI clinic urbanity.

**Results:**

FGM FSW (*n* = 5085) mostly originated from Eastern Europe (50.5 %) and SGM FSW (*n* = 1309) from Suriname/Netherlands Antilles (36.3 %). Among FGM, SGM and Western-born FSW, 11.4 %, 15.2 % and 13.3 %, respectively (*p* < 0.001) were diagnosed with any STI. FGM FSW had a lower odds (aOR 0.78, 95 %:CI 0.65–0.94,*p* < 0.01) of chlamydia or gonorrhoea diagnosis, but a higher aOR (6.38,95 %CI:2.63–15.49,*p* < 0.001) of HIV, syphilis, or hepatitis B diagnosis in the first consultation. FGM FSW had a lower likelihood of a repeat consultation at any time (aHR:0.73,95 %CI:0.69–0.77,*p* < 0.001) than Western-born FSW.

**Conclusion:**

Migrant FSW versus Western-born FSW demonstrated a varying burden of STI, FGM heightened proportions and odds of infectious syphilis, hepatitis B and HIV and lower likelihood of repeat consultations. Enhancing accessibility and outreach efforts for migrant FSW in sexual healthcare services is imperative.

## Highlights

• Largest cohort study examining migrant FSW STI burden and healthcare engagement.
• Migrant FSW have higher burden and odds of infectious syphilis, hepatitis B, and HIV.
• Western-born FSW have higher burden of CT and NG than first-generation FSW.
• First-generation migrants less likely to have a repeat consultation versus Western-born.
• Targeted outreach needed in STI clinics to improve migrant FSW healthcare retention.

## 1 Introduction

There has been an increase of migration of sex workers to and within Europe over the years (TAMPEP, 2015). The European sex work population still largely constitutes of women, of which almost half (47 %) are migrants (TAMPEP, 2009). The growing overall migrant influx in Europe has been a crisis situation since 2015 (Eurostat 2023). Over the years, millions of people migrated to Europe from war, geopolitical unrest and economic hardship in their home country. In 2022, 23·8 million and 5·3 % of the total European Union (EU) population were non-EU citizen (Eurostat 2023). Adequate access to healthcare is crucial for migrants arriving in (Western) Europe, as migration-related factors such as poor living conditions, changes in lifestyle and a lack of access to (health) services make migrants more vulnerable for health conditions (WHO 2023). European action pillars to improve migrant health include the development of inclusive environments that promote public health, social inclusion and well-being and strengthening of evidence- and data-driven policy-making (WHO 2023).

Migrant female sex workers (FSW) are considered a more vulnerable group than non-migrant FSW, with their work and life circumstances both increasing their likelihood of an sexually transmitted infections (STI) and limiting their healthcare-seeking behaviour (Cwikel et al., 2008). Migration has shown to result into health and structural vulnerabilities for FSW, such as condom refusal by clients and experiencing barriers to accessing healthcare (Goldenberg et al., 2014). A systematic review established that in high-income countries, migrant FSW have a higher likelihood of STI, but a lower likelihood of human immunodeficiency virus (HIV) compared to non-migrant FSW, although only nine mostly small European studies could be included (Platt et al., 2013). Migrant FSW's access to sexual healthcare is hampered by structural determinants, e.g. language barriers, (HIV) stigma and low sexual health knowledge and risk perception, migration and sex work policies, and intermediary determinants, e.g. health system navigation, lack of trust in healthcare provider and negative experiences in accessing healthcare services in their home country (Tokar et al., 2020; Parra et al., 2024).

A Spanish study showed that migrant FSW significantly more often had never accessed sexual health services and had not been tested for HIV compared to non-migrant FSW (Dias et al., 2017). On the other hand, a study from the United Kingdom (UK) established no significant difference in use of sexual healthcare services, i.e. STI/HIV testing, between Eastern European FSW and UK-born FSW (Platt et al., 2011). Additionally, a German study presented differences in general healthcare utilisation among first- and second-generation migrants and native-borns, but this phenomenon has not been studied before in (female) sex workers in Western Europe (Glaesmer et al., 2011). European studies thus remain inconclusive on the impact of migration on the healthcare-seeking behaviour of migrant FSW.

Previous studies highlighted a need for monitoring and research on the likelihood of STI for migrant FSW and how it differs from local, non-migrant FSW (Platt et al., 2013). Available studies on this topic from high-income countries, and more specifically from Western Europe, however remain limited. Most studies are small, not recent and none of the studies have made a distinction between first- and second-generation migrants.

Therefore, this study aims to evaluate the representation of migrant FSW in all sexual healthcare-consultations of all Dutch STI clinics. It will assess sociodemographic characteristics, likelihood and burden of STI/HIV and engagement in sexual healthcare among first- and second-generation migrant and Western-born FSW. The results of this study aim to inform STI clinics and evidence-driven public health policy.

## 2 Material and methods

### 2.1 Study design

In this retrospective cohort study, coded surveillance consultations of FSW were included from all outpatient Public Health STI clinics in the Netherlands (25 Public Health Services with 38 STI clinic locations) which were submitted between January 1st 2016 and December 31st 2021 via an electronic patient registry using a consultation code, to the National Institute of Public Health and the Environment. The publicly funded STI clinics are required to submit anonymised consultations to the national institute.

Dutch STI clinics provide free of charge, anonymous and confidential sexual healthcare to high-incidence STI groups including, among others, sex workers and individuals from STI-endemic countries. All FSW are routinely tested for urogenital, anorectal, and oropharyngeal Chlamydia trachomatis (CT) and Neisseria gonorrhoea (NG), HIV, hepatitis B and infectious syphilis. Self-collected vaginal and anorectal swabs and self- or provider-collected oropharyngeal swabs were tested for CT and NG. Blood samples were tested for HIV, hepatitis B and infectious syphilis. The specimens were processed at different Dutch medical microbiological laboratories using commercially available diagnostic tests. After hepatitis B vaccination, testing for hepatitis B is omitted. Consultation-level and individual-level data was extracted on sociodemographic characteristics, sexual behaviour and STI diagnoses. Sociodemographic characteristics and sexual behaviour were obtained from a medical and sexual history patient questionnaire conducted by STI clinic nurses and registered in the electronic patient registry.

Of all consultations included in the surveillance data from the years 2016 – 2021 (*n* = 799,400), those who were not from FSW were excluded. Finally, 27,532 consultations from FSW were included in this study.

### 2.2 Study context

In 2022, 25·9 % of the in total 17·7 million Dutch inhabitants had a migration background, of which 14 % was not born in the Netherlands (CBS, 2022b). The main countries of origin from Dutch inhabitants with a migration background were Turkey, Morocco, Suriname, Indonesia, Germany and Poland. (CBS, 2022b) Suriname, Indonesia and the Netherlands (NL) Antilles are former Dutch colonies and Turkey and Morocco are former guest workers countries (de Regt and Tineke, 2022). The islands that were part of the Netherlands Antilles are nowadays part of the Dutch Kingdom as separate countries or as special municipalities.

Estimates of the (female) sex worker population in the Netherlands vary. UNAIDS data from 2011 estimated the total sex worker population to be 25,000 (UNAIDS, 2023) while other research estimated 0·6 % of the Dutch female population to be sex worker (Vandepitte et al., 2006), resulting into an estimate of 53,765 FSW in 2023. In 2000, the brothel ban was lifted and sex work was legalised and recognised as labour in the Netherlands. Only EU citizen are permitted to work in sex work, non-EU citizen are not able to obtain a work permit for sex work (TAMPEP, 2009).

### 2.3 Definitions

FSW are defined as women who have sex in exchange for money or goods. Western-born is defined as born in Northern, Central, Southern and Western Europe, Oceania or North America. While we are aware that the terms immigrant and migrant are both used in literature and have a partly overlapping meaning, we choose to use the broader term migrant. First-generation migrant is defined as born in Eastern Europe, Latin America, Asia, Africa, Netherlands Antilles or Suriname. Second-generation migrant is defined as Western-born with at least one parent who is born in Eastern Europe, Latin America, Asia, Africa, Netherlands Antilles or Suriname. Level of education was categorized into low: elementary, pre-vocational secondary; medium: senior general secondary, pre-university, secondary vocational; high: higher professional, university based on the definitions used by Statistics Netherlands (CBS, 2022a).

Urbanity of STI clinic region was assigned to the STI clinic region based on number of inhabitants per KM^2^ in the region according to Statistics Netherlands. (CBS, 2022a) If an STI clinic region had ≥ 900 inhabitants per KM^2^, the region was assigned ‘High urban’ and if the region had < 900 inhabitants per KM^2^ the region was assigned ‘Moderate to low urban’.

Condom use during anogenital sex has a ‘not applicable’ option, since some FSW do not have anogenital sex. For alcohol/drug use during sex <6 months the options ‘no’ and ‘unknown’ were merged together into ‘no’.

Burden of STI/HIV was measured with STI diagnoses, which was defined as being diagnosed with CT, NG, infectious syphilis (primary syphilis, secondary syphilis and early latent syphilis), infectious hepatitis B and/or a newly diagnosed HIV infection in the first consultation or the first repeat consultation of the study period. Healthcare seeking behaviour can be challenging to measure, especially since there is no national data available on the number of (migrant) sex workers. Healthcare engagement is a more accessible metric to assess accessibility of healthcare. Thus, this study will use retest behaviour, i.e. repeat consultations of FSW, as a measure for (continued) sexual healthcare engagement. Engagement in sexual healthcare was measured by assessing a repeat consultation on an individual-level. This included the first STI test consultation one month after the first STI test consultation in the available data carried out by the same STI clinic. We excluded all consultations within one month after the previous consultation, to ensure that the consultation is not merely a follow-up to the previous consultation.

### 2.4 Data analyses

Descriptive analyses were performed to describe proportions of sociodemographic characteristics, sexual behaviour, STI diagnoses in the first consultation, among continents and for one-time and repeat testers among first- and second-generation migrant and Western-born FSW.

Sociodemographic characteristics and STI diagnoses were compared between first- and second-generation migrant and Western-born FSW using chi-square tests. If more than 2·5 % of values was missing, a category ‘unknown’ was created and reported in the results.

A logistic regression analysis was performed to test associations between migration and STI diagnoses in the first consultation while adjusting for age, urbanity of the STI clinic region and sexual behaviour. Sexual behaviour included the variables number of sex partners, sex with and alcohol/drug use during sex <6 months. Condom use during anogenital sex and PrEP use were not adjusted for as sexual behaviour, since they were not available for the entire study period but from 2018 onwards. For the regression analysis, the STI chlamydia and gonorrhoea were combined and new HIV, infectious syphilis and infectious Hepatitis B were combined.

A Kaplan-Meier survival plot was performed to compare the probability of having a (first) repeat consultation among the three FSW groups (first-generation, second- generation migrant and Western-born FSW) over the study years, with a log-rank test comparing the three survival curves. The primary outcome was the occurrence of a first repeat consultation after the initial STI clinic visit during the study period. Exposure time was defined as the time between the first consultation until the first repeat consultation or the end of the study period.

A Cox proportional hazard regression was performed to assess the risk of having a repeat consultation for first- and second-generation FSW compared to Western-born FSW groups, adjusting for age, urbanity and STI diagnoses in the first consultation. The assessed hazard ratio (HR) showed the probability of a repeat consultation happening among first-generation migrant FSW and second-generation migrant FSW relative to the probability of a repeat consultation among Western-born FSW over the years. An alpha of ≤0·01 was considered significant in all analyses due to the large sample size. Statistical analyses were done using IBM SPSS Statistics (Version 27·0, Armonk, NY, USA).

### 2.5 Ethical considerations

The Medical Ethics Committee of Maastricht University waived the requirement for ethical approval and written informed consent because the data used were coded, originated from standard care, and were analysed anonymously (METC 2017–0251).

## 3 Results

This study included 27,532 STI clinic consultations of 11,363 unique FSW. Of this group of FSW, 5085 were first-generation migrants, 1309 were second-generation migrants and 4969 were Western-born. Sociodemographic characteristics of the study population are presented in Table 1. The median age of FSW was lower among the second-generation migrants in comparison to first-generation migrant and Western-born FSW (*p* < 0·001). The level of education was unknown for a greater part (40·5 %) of the first-generation migrants and as well as for the second-generation migrant (12·7 %) and Western-born (19·8 %) FSW (*p* < 0·001). A large proportion of the first-generation migrant FSW was born in Eastern Europe (50·5 %), followed by Latin America (27·2 %) and Asia (9·9 %), while the birth region of the second-generation migrant FSW's parent(s) was mostly Suriname/Netherlands Antilles (36·3 %), followed by North Africa (25·7) and Asia (15·7 %). More first- and second-generation migrant FSW visited STI clinics in a high urban region compared to Western-born FSW (*p* < 0·001).

**Table 1:** Sociodemographic characteristics of the study population of (migrant) female sex workers (FSW) who visited an STI clinic from 2016 to 2021 in The Netherlands (*n* = 11,363).

	First-generation migrant FSW *N* = 5085 % (N)	Second-generation migrant FSW *N* = 1309 % (N)	Western-born FSW *N* = 4969 % (N)	Chi-square test P-value
**All consultations**	41·4 (11,390)	11·4 (3147)	47·2 (12,995)	–
**Unique persons**	44·8 (5085)	11·5 (1309)	43·7 (4969)	–
**Sociodemographic characteristics of first consultation**				
Age in years (median; IQR)	32 (26 – 41)	27 (23 – 33)	31 (25 – 42)	**<0·001**^1^
Level of education				
High	7·7 (390)	24·4 (319)	27·3 (1356)	**<0·001**
Middle	6·2 (317)	16·9 (221)	12·9 (642)	
Low	21·7 (1102)	43·5 (569)	37·4 (1859)	
Different	24·0 (1219)	2·6 (34)	2·6 (130)	
Unknown	40·5 (2057)	12·7 (166)	19·8 (982)	
Birth region FSW's parent(s)				n/a
Eastern Europe	50·5 (2567)	10·0 (131)		
Latin America	27·2 (1383)	9·8 (128)		
Asia	9·9 (502)	15·7 (206)		
Sub-Sahara Africa	5·6 (286)	8·6 (112)		
Suriname/Netherlands Antilles	5·1 (258)	36·3 (475)		
North Africa	1·8 (89)	25·7 (337)		
Urbanity of STI clinic region				
High urban	68·3 (3473)	71·5 (936)	49·8 (2476)	**<0·001**
Moderate to low urban	31·7 (1612)	28·5 (373)	50·2 (2493)	

1 Anova to compare means.

The study population's sexual behaviour as reported in the first consultation and diagnoses of STI in the first consultation are presented in Table 2.

**Table 2:** Sexual behaviour and sexually transmitted infections (STI) diagnoses in first consultation of (migrant) female sex workers (FSW) who visited an STI clinic from 2016 to 2021 in The Netherlands (*n* = 11,363).

	First-generation migrant FSW *N* = 5085 % (N)	Second-generation migrant FSW *N* = 1309 % (N)	Western-born FSW *N* = 4969 % (N)	Chi-square test P-value
**Sexual behaviour**				
Number of sex partners <6 months (median; IQR)	100 (50 – 999)	50 (10 – 200)	40 (10 – 999)	**<0·001**^3^
Condom use during anogenital sex^1^				
N/A	0·5 (12)	0·3 (2)	0·9 (20)	**<0·001**
Never	10·5 (231)	14·6 (94)	15·8 (368)	
Not always	42·2 (931)	56·4 (364)	54·8 (1279)	
Always	46·8 (1034)	28·7 (185)	28·6 (669)	
PrEP use^1^^,^^2^				
No	99·96 (2255)	100 (652)	99·96 (2368)	0·868
Yes	0·04 (1)	0 (0)	0·04 (1)	
Sex with				
Men	93·9 (4774)	80·0 (1047)	66·9 (3324)	**<0·001**
Women	0·5 (27)	0·4 (5)	0·6 (28)	
Men and women	5·6 (283)	19·6 (257)	32·5 (1616)	
Alcohol/drug use during sex <6 months				
No/unknown	89·8 (4568)	72·7 (952)	73·4 (3646)	**<0·001**
Yes	10·2 (517)	27·3 (357)	26·6 (1323)	
**STI**				
Any STI				**<0·001**
Yes	11·4 (580)	15·2 (199)	13·3 (661)	
No	88·6 (4505)	84·8 (1110)	86·7 (4308)	
New HIV infection				
Yes	0·2 (12)	0·0 (0)	0.1(3)	0·020
No	99·8 (5073)	100,309)	99·9 (4966)	
Infectious syphilis				
Yes	0·5 (23)	0.2 (2)	0·1 (4)	**<0·001**
No	97·8 (4973)	97·4 (1275)	97·1 (4825)	
Not tested	1·8 (89)	2·4 (32)	2·8 (140)	
Infectious hepatitis B				
Yes	0·8 (41)	0·2 (3)	0·0 (2)	**<0·001**
No	53·6 (2728)	57·8 (756)	48·7 (2418)	
Not tested	45·5 (2316)	42·0 (550)	51·3 (2549)	
Chlamydia				
Yes	7·3 (373)	11·8 (154)	10·2 (505)	**<0·001**
No	92·5 (4706)	87·9 (1151)	89·7 (4457)	
Not tested	0·1 (6)	0·3 (4)	0·1 (7)	
Gonorrhoea				
Yes	3·4 (172)	4·3 (56)	4·1 (203)	0·112
No	96·6 (4906)	95·7 (1249)	95·9 (4759)	
**Diagnoses of any STI within geographical regions**^4^				
Sub-Saharan Africa	15·0 %	11·7 %	–	–
Suriname/NL Antilles	13·4 %	15·3 %	–	–
Asia	12·5 %	16·7 %	–	–
Eastern Europe	12·0 %	9·2 %	–	–
North Africa	11·2 %	17·1 %	–	–
Latin America	8·7 %	16·4 %	–	–

1 Available from 2018 and onwards.

2 Reported in the last consultation, instead of the first consultation.

3 Anova to compare means.

4 STI burden significantly differed between geographical regions among the first-generation migrant FSW (*p* = 0·006) but not among the second-generation migrant FSW (*p* = 0·318).

First-generation migrant FSW had a higher interquartile range (IQR) of sex partners in the past 6 months in comparison to second-generation migrant and Western-born FSW (*p* < 0·001). The majority of first- and second generation migrant FSW had sex with men (93·3 %; 80 %), while part (32·5 %) of the Western-born FSW had sex with both women and men (*p* < 0·001). Use of substances (alcohol/drugs) was lowest among first-generation migrant FSW (*p* < 0·001) and this group most often reported to ‘always’ use a condom during anogenital sex (*p* < 0·001).

There was a significant difference in diagnoses of any STI (11·4 %, 15·2 %, 13·3 %; *p* < 0·001), infectious syphilis (0·5 %; 0·2 %; 0·1 %; *p* < 0·001), infectious hepatitis B (0·8 %, 0·2 %, 0·0 %; *p* < 0·001), and chlamydia (7·3 %, 11·8 %, 10·2 %; *p* < 0·001) between the first-generation migrant, second-generation migrant and Western-born FSW, respectively.

Between geographical regions, first-generation migrant FSW from Sub-Sahara Africa had the highest burden of any STI (15·0 %), followed by Suriname/NL Antilles (13·4 %) and Asia (12·5 %) (*p* = 0·006). Second-generation migrant FSW from North Africa had the highest burden of any STI (17·1 %), followed by FSW from Asia (16·7 %) and Latin America (16·4 %) (*p* = 0·318). Burden of any STI were differed significantly between geographical regions among first-generation migrant FSW (*p* = 0·006).

The association between diagnoses of STI in first consultation and migrant group is presented in Table 3. When adjusting for age and urbanity of the STI clinic region, first-generation migrant FSW (in reference to Western-born FSW) have an aOR of 0·76 (95 %CI 0·67 – 0·86, *p* < 0·001) of a chlamydia or gonorrhoea diagnosis and an aOR of 9·25 (95 %CI 4·60 – 18·60, *p* < 0·001) of a new HIV, infectious syphilis or infectious hepatitis B diagnosis in the first consultation. When adjusting for age, urbanity of the STI clinic region and sexual behaviour, first-generation migrant FSW (in reference to Western-born FSW) have an aOR of 0·78 (95 %CI 0·65 – 0·94, *p* < 0·01) of a chlamydia or gonorrhoea diagnosis and an aOR of 6·38 (95 %CI 2·63 – 15·49, *p* < 0·001) of a new HIV, infectious syphilis or infectious hepatitis B diagnosis in the first consultation.

**Table 3:** Association between diagnoses of sexually transmitted infections (STI) in first consultation and FSW migrant groups who visited an STI clinic from 2016 to 2021 in The Netherlands.

Main determinant	OR (95 % CI)	aOR^1^ (95 % CI)	aOR^2^ (95 % CI)
**Chlamydia & gonorrhoea**			
First-generation migrant FSW	0·74 (0·66 – 0·84)***	0·76 (0·67 – 0·86)***	0·78 (0·65 – 0·94)**
Second-generation migrant FSW	1·15 (0·97 – 1·37)	0·99 (0·84 – 1·19)	1·07 (0·85 – 1·35)
Western-born FSW	1 (ref)	1 (ref)	1 (ref)
**New HIV, infectious syphilis & infectious hepatitis B**			
First-generation migrant FSW	8·14 (4·07 – 16·28)***	9·25 (4·60 – 18·60)***	6·38 (2·63 – 15·49)***
Second-generation migrant FSW	2·11 (0·71 – 6·32)	2·61 (0·86 – 7·89)	0·64 (0·77 – 5·30)
Western-born FSW	1 (ref)	1 (ref)	1 (ref)

1 Adjusted for age and urbanity of STI clinic region.

2 Adjusted for age, urbanity of STI clinic region and sexual behaviour *p < 0·05, **p < 0·01, *** *p* < 0·001.

Incidence of a first repeat consultation was 17·2 per 100 person-years (95 %CI 16·6–18·0) among first-generation migrant FSW, 21 per 100 person-years (95 %CI 19·4–22·8) among second-generation migrant FSW and 25·8 (95 %CI 24·8–26·9) among Western-born FSW.

**Fig. 1:**
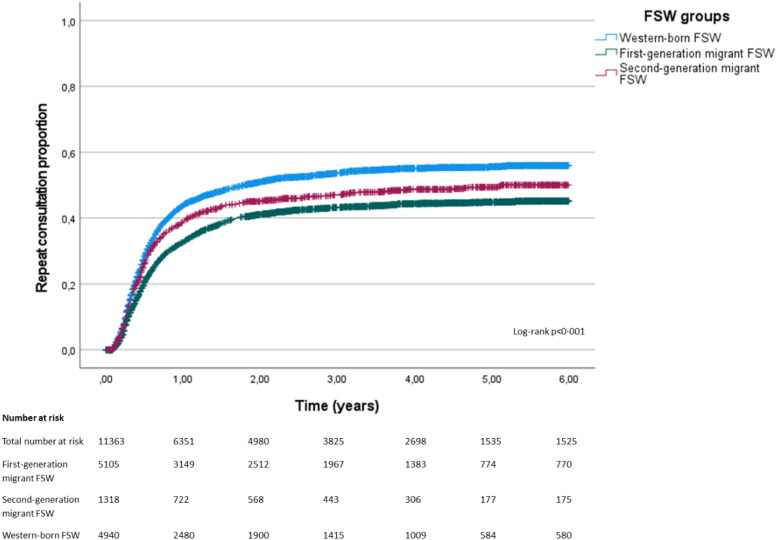
Survival curve of the proportion of repeat consultations in the years 2016 to 2021 among first-, second-generation and Western-born female sex workers (FSW) (*n* = 11,363).

In Fig. 1, Kaplan-Meier survival curves of repeat consultations among the FSW groups over the years 2016 – 2021 are presented. The log-rank test indicated that the survival curves were significantly different (*p* < 0.001). First-generation migrant FSW (in reference to Western-born FSW) have an aHR of 0·73 (95 %CI 0·69 – 0·77, *p* < 0·001) of having a (first) repeat consultation at any time. Second-generation migrant FSW (in reference to Western-born FSW) have a (borderline significant) aHR of 0·89 (95 %CI 0·81 – 0·97, *p* = 0·011) of having a (first) repeat consultation at any time.

STI/HIV burden for one-time testers and repeat testers among first-, second-generation and Western-born FSW are presented in Appendix Table A.1. Diagnoses of any STI decreased in the first repeat consultation compared to the first consultation of first- (*p* < 0·001) and second-generation (*p* = 0·005) migrant and Western-born FSW (*p* = 0·077) repeat testers. Chlamydia diagnoses significantly decreased in the first repeat consultation compared to the first consultation for first-generation migrant (*p* < 0·001) and Western-born (*p* = 0·002) FSW repeat testers.

## 4 Discussion

This study assessed sociodemographic characteristics, burden and likelihood of STI/HIV and engagement in sexual healthcare in a large population of first- and second-generation migrant and Western-born FSW in the Netherlands. The calculated burden of any STI was slightly higher among second-generation FSW with 11·4 %, 15·2 % and 13·3 % (*p* < 0·001) respectively. The burden of newly diagnosed HIV, infectious syphilis and infectious hepatitis B was highest among first-generation migrant FSW, while the burden of chlamydia and gonorrhoea was highest among second-generation migrant FSW followed by Western-born FSW. Furthermore, first-generation migrant FSW have a lower odds of a chlamydia or gonorrhoea diagnosis (aOR 0.78, 95 %CI 0.65 – 0.94, *p* < 0.01) and a higher odds of a new HIV, infectious syphilis or infectious hepatitis B diagnosis (aOR 6.38, 95 %CI 2.63 – 15.49, *p* < 0·001) in the first consultation when adjusting for age, urbanity of the STI clinic and sexual behaviour. A sensitivity analysis adjusting for all sexual behaviour including the variables condom use during anogenital sex and PrEP use in study data from 2018 and onwards yielded comparable results, confirming the robustness of these findings.

When comparing geographical regions within the migrant groups, first-generation migrants from Sub-Saharan Africa and second-generation migrants from North Africa had the highest burden of STI. First-generation migrant FSW had a lower likelihood of a first repeat consultation at any time (HR 0·73 (0·69–0·77, *p* < 0·001) in comparison to Western-born FSW.

The scale and scope of this study represents a significant contribution to the research field, however its uniqueness also complicates comparison to the limited existing literature.

In this study, the HIV burden did not significantly differ among first- and second-generation migrant and Western-born FSW (0·2 %, 1·0 % and 0·3 % respectively, *p* = 0·020). Platt et al. established a higher HIV burden among migrant and non-migrant FSW in high-income countries (4 % and 4·5 % respectively) and a substantially lower STI prevalence (5 % and 3 % respectively) compared to this study (Platt et al., 2013). Additionally, their calculated STI burden was lower among non-migrant FSW versus migrant FSW, contrary to the finding of this study (Platt et al., 2013).

Interestingly, a comparable study was conducted by our research group among male sex workers who have sex with men (MSW-MSM), in which inverse results were determined to those in this study. First-generation migrant MSW-MSM had the highest burden of STI (33·2 %), compared to second-generation (29·3 %) and Western-born MSW-MSM (23·3 %) (Peters et al., 2024). Furthermore, first-generation migrant MSW-MSM were more likely to have a repeat consultation (aHR 1·5, 95 %CI 1·3–1·8) compared to Western-born MSW-MSM (Peters et al., 2024). Possibly, first-generation migrant FSW are more mobile than their MSW-MSM counterparts, as a previous Dutch study describes testimonials from migrant FSW going on ‘tours’ (Tokar et al., 2020). Subsequently, they may visit an STI clinic in another region in which they will be registered as a new patient and thus no repeat consultation is recorded. Migrant FSW in Ireland also stated that their constant mobility affects their (sexual) health and causes them to remain invisible for health services (Sweeney and FitzGerald, 2017). In general, migrant FSW seem less likely to engage in (sexual) healthcare or to be registered with a general practitioner (GP) (Platt et al., 2011; Richter et al., 2014). This may also reflect on migrant sex workers facing barriers to access sexual healthcare services, as previously identified in a Dutch study examining HIV testing access for migrant FSW (Tokar et al., 2020).

Both migrant MSW-MSM and FSW studies have identified higher percentages of diagnoses with the more severe STI (HIV, infectious hepatitis B and infectious syphilis) among first- and second-generation migrants compared to Western-born, although percentages are low (Peters et al., 2024). Specifically, first-generation migrant FSW in this study had the highest burden and a higher odds of these severe STI, while their burden and odds of chlamydia and gonorrhoea was lower. This suggests that different behavioural and risk factors and social determinants of health are at play within this migrant group, such as variations in sexual network and sexual health(care) knowledge and access and cultural practices, which warrants further research and highlights the need for tailored STI prevention strategies.

In this study, we also established a significant difference in diagnoses with any STI between geographical regions within the group of first-generation migrant FSW, contrary to a Spanish study concluding that STI prevalences of migrant FSW were not significantly different by geographical origin (del Amo et al., 2005).

Self-reported sexual behaviour in this study all, except for PrEP use, showed significant differences among the FSW groups. First-generation migrant FSW reported the highest number of sex partners in the past 6 months, the highest proportion of ‘always’ using a condom during anogenital sex and the lowest proportion of alcohol/drug use during sex in the past 6 months. These self-reported behaviours could possibly indicate more professionalisation of sex work among the first-generation FSW. A UK study found similar results of migrant FSW having a higher number of sex work clients, and a lower proportion of non-condom use and alcohol/drug use compared to non-migrant FSW (Platt et al., 2011). Migrant FSW's motivation for the entry of sex work was also more often to save or support studies and less often related to survival, debts or drugs than for non-migrant FSW, suggesting lower vulnerability among migrant FSW (Platt et al., 2011).

### 4.1 Strengths and limitations

To our knowledge, this is the first study in Western Europe comparing STI/HIV burden and sexual healthcare engagement of first-generation and second-generation migrant and Western-born FSW. Additionally, the long study period from 2016 to 2021 allowed us to include large cohort of 27,532 STI clinic consultations from 11,363 unique FSW. While using this systematically collected data provides valuable real-world insights, the electronic patient registry data also has limitations. First, due to the selected inclusion period of the data we might have introduced classification bias as a first STI consultation of a FSW could possibly be a repeat consultation. Second, the number of unique patients might be an overestimation since a patient will be registered as a new patient at a different STI clinic region and could have multiple files per clinic due to the ability to register anonymously. Third, since STI such as HIV and syphilis can remain undetected for extended periods, the period during which STI were acquired may be long before the first consultation compared to the repeat consultation. This time discrepancy between infection acquisition and diagnosis is a limitation in our approach of comparing first and repeat consultations and should be understood when interpreting our study findings.

Fourth, we were unable to adjust for certain sociodemographic and behavioural characteristics in some analyses due to unknown/missing data. However, a sensitivity analysis including all sexual behaviours was performed to ensure the robustness of the logistic regression associations.

Fifth, we do not have data on STI consultations at other healthcare providers, such as GPs, which limits our study's generalisability. Sixth, the limited variables available in our dataset hinder our ability to explain the observed STI burden among migrant FSW. A deeper understanding of the complex interplay of social determinants of health influencing migrants' STI risk and sexual health is essential to inform effective prevention strategies and sexual healthcare interventions.

### 4.2 Implications for care

It could be hypothesized that part of the migrant FSW have not yet visited the STI clinic, either due to experiencing barriers, or due to being unaware of the services available. It seems that those migrant FSW who do come into care, are also more often lost to care in comparison to Western-born FSW. This applies to both first- and second-generation migrant, while first-generation FSW do have an even lower incidence of a repeat consultation per 100 person years (17·2 versus 21). This phenomenon could partly be explained by migrant FSW their ‘touring’ behaviour (Tokar et al., 2020). On the other hand, it is deemed essential that sexual health service providers make their services accessible for migrant FSW, are stigma-informed services and meet the needs of migrant FSW (Sweeney and FitzGerald, 2017). STI clinics should expand their outreach services in an effort to reach migrant FSW with their sexual healthcare services. Building trust and providing social support could improve retained engagement in sexual healthcare services of migrant FSW (Tokar et al., 2020).

## 5 Conclusion

This unique and large study shows that first- and second-generation migrant FSW compared to Western-born FSW have a varying burden of any STI and have a higher burden of infectious syphilis, infectious hepatitis B and HIV. Furthermore, first-generation migrant FSW have a lower odds of a chlamydia or gonorrhoea diagnosis but a higher odds of a new HIV, infectious syphilis or infectious hepatitis B diagnosis.

Nevertheless, first- and second-generation migrant FSW had lower incidence of a (first) repeat consultations and first-generation migrant FSW were less likely to have a repeat consultation than Western-born FSW. These findings implicate that first-generation migrant FSW are more lost to follow-up in STI clinic care compared to Western-born FSW. Sexual healthcare services such as STI clinics should enhance their accessibility migrant FSW, amongst others, by addressing experienced barriers and incorporating their healthcare needs. Furthermore, STI clinics should intensify their outreach efforts on reaching migrant FSW and retaining them in care in order to decrease their burden of severe STI. Future research should focus on co-creating targeted intervention programs for migrant FSW to mitigate disparities between migrant and non-migrant FSW.

## Data sharing

The data used were explicitly made available for this study by the National Institute for Public Health and the Environment. Any data sharing requests should be directed to the National Institute for Public Health and the Environment (soap@rivm.nl).

## Ethical considerations

The Medical Ethics Committee of Maastricht University waived the requirement for ethical approval and written informed consent because the data used were coded, originated from standard care, and were analyzed anonymously (METC 2017–0251).

## Role of the funding source

This study is an investigator initiated study and was funded by the employer of the main investigator. The funder of the study had no role in the study design, data collection, data analysis, data interpretation, and writing of the manuscript.

## CRediT authorship contribution statement

**C.M.M. Peters:** Writing – review & editing, Writing – original draft, Visualization, Software, Investigation, Formal analysis, Data curation, Conceptualization. **Y.J. Evers:** Writing – review & editing, Validation, Supervision, Methodology, Investigation, Formal analysis, Data curation, Conceptualization. **C.J.G. Kampman:** Writing – review & editing, Writing – original draft. **M.J. Theunissen–Lamers:** Writing – review & editing. **M.A.M. van den Elshout:** Writing – review & editing. **N.H.T.M. Dukers-Muijrers:** Writing – review & editing, Supervision, Methodology, Investigation, Data curation, Conceptualization. **C.J.P.A. Hoebe:** Writing – review & editing, Validation, Supervision, Methodology, Investigation, Data curation, Conceptualization.

## Declaration of competing interest

The authors declare that they have no known competing financial interests or personal relationships that could have appeared to influence the work reported in this paper.

## Appendix Supplementary materials

Image, application 1
