# Supplementary material for: Engagement in sexual healthcare and STI/HIV burden of first- and second-generation migrant and Western-born female sex workers in the Netherlands: A retrospective cohort study
Source: J Migr Health. 2024 Oct 31;10:100281. doi: 10.1016/j.jmh.2024.100281 (PMC11570462; doi:10.1016/j.jmh.2024.100281)
Supplement: Supplementary file 1 [file mmc1.docx]

**8. Appendices**

**Appendix Table A.1. STI/HIV burden for one-time testers and repeat testers among first- and second generation and Western-born female sex workers (FSW) who visited an STI clinic from 2016 to 2021 in The Netherlands (n=11363)**

|  | **First generation migrants**  **N=5085** | | | | **Second generation migrants**  **N=1309** | | | | **Western born**  **N=4969** | | | |
| --- | --- | --- | --- | --- | --- | --- | --- | --- | --- | --- | --- | --- |
|  | **One-time testers**  **% (N)** | **Repeat testers**  ***% (N)*** | | | **One-time testers**  **% (N)** | **Repeat testers**  **% (N)** | | | **One-time testers**  **% (N)** | **Repeat testers**  **% (N)** | | |
|  |  | *1st consultation* | *1st repeat consultation* | *P-value (McNemar)* |  | *1st consultation* | *1st repeat consultation* | *P-value (McNemar)* |  | *1st consultation* | *1st repeat consultation* | *P-value (McNemar)* |
| Any STI | 11.9% (358) | 10.6% (222) | 7.3% (153) | **<0.001** | 14.9% (108) | 15.6% (91) | 10.5% (62) | **0.005** | 13.3% (325) | 13.3 (336) | 11.7 (292) | 0.077 |
| New HIV infection | 0.4% (12) | 0.0% (0) | 0.0% (0) | n/a | 0.0% (0) | 0.0% (0) | 0.0% (0) | n/a | 0.1% (3) | 0.0% (0) | 0.0% (1) | n/a |
| Infectious syphilis | 0.7% (20) | 0.1% (3) | 0.0% (0) | n/a | 0.1% (1) | 0.2% (1) | 0.0% (0) | n/a | 0.0% (1) | 0.1% (3) | 0.1% (3) | n/a |
| Infectious hepatitis B | 1.2 % (35) | 0.3 (6) | 0.1 (2) | n/a | 0.3% (2) | 0.2 (1) | 0.0% (0) | n/a | 0.0% (0) | 0.1 (2) | 0.0% (1) | n/a |
| Chlamydia | 7.1% (213) | 7.7% (160) | 5.2% (109) | **<0.001** | 11.6% (84) | 12% (70) | 8.1% (48) | 0.017 | 10.0% (245) | 10.3% (260) | 7.8% (194) | **0.002** |
| Gonorrhoea | 3.4% (103) | 3.3% (69) | 2.1% (45 | 0.021 | 3.6% (26) | 5.1 (30) | 3.4 (20) | 0.165 | 4.2% (102) | 4.0% (101) | 4.4% (110) | 0.552 |
